# Association of DNA damage repair related genes with prostate cancer: A multi-omics Mendelian Randomization analysis

**DOI:** 10.1097/MD.0000000000049185

**Published:** 2026-05-29

**Authors:** Huadong Xie, Chengjie Ban, Zhengwei Su, Qingming Huang, Han Tang, Zhi Cheng, Tianling Liao, Kangji Liao, Xianlin Yi

**Affiliations:** a Department of Urology, Hubei Province Human Sperm Bank, Maternal and Child Health Hospital of Hubei Province, Tongji Medical College, Huazhong University of Science and Technology, Wuhan, Hubei, China; b Department of Urology, Liuzhou Workers’ Hospital, Liuzhou, Guangxi, China; c Department of Urology, The First Affiliated Hospital of Guangxi University of Chinese Medicine, Nanning, Guangxi, China; d School of Graduates, Guangxi Medical University, Nanning, Guangxi, China; e Guangxi Key Laboratory of Extremely Weak Magnetic Field in Cancer Medicine, Guangxi Medical University Cancer Hospital, Nanning, Guangxi, China; f Guangxi Clinical Research Center for Bladder Cancer, Guangxi Medical University Cancer Hospital, Nanning, Guangxi, China; g University Engineering Research Center of Oncolytic & Nanosystem Development, Guangxi Medical University, Nanning, Guangxi, China.

**Keywords:** DNA damage repair, gene expression, gene methylation, Mendelian Randomization, prostate cancer, protein abundance

## Abstract

The relationship between prostate cancer and DNA damage repair remains incompletely elucidated. This study employs Mendelian Randomization to explore the causal relationships between DNA Damage Repair-related gene methylation, expression, and protein abundance and prostate cancer risk. A comprehensive analysis was conducted on 2736 DNA Damage Repair-related genes identified from the GeneCards database using Summary-data-based Mendelian Randomization. The study utilized quantitative trait loci datasets from multiple European cohorts, alongside Genome-Wide Association Studies data. Candidate gene expression patterns in prostate cancer tissue were further examined using the Human Protein Atlas and Tumor Immune Single-cell Hub 2 databases. Summary-data-based Mendelian Randomization analysis identified significant associations of genetically predicted RPA2 and POLI with prostate cancer risk. Immunohistochemical analyses from the human protein atlas database confirmed RPA2 and POLI expression in prostate cancer tissue, while single-cell sequencing data from the tumor immune single-cell hub 2 database indicated differential expression across distinct cellular subpopulations. Our study reveals a genetically inferred causal role for DDR-related genes RPA2 and POLI in prostate cancer risk. These findings may enhance the understanding of the molecular mechanisms linking DNA Damage Repair-related genes and prostate cancer.

## 1. Introduction

Prostate cancer (PCa) remains one of the most prevalent malignancies among men globally, with several factors, such as age, family history, ethnicity, hormonal influences, and specific genetic mutations, contributing to its development.^[1–3]^ Annually, over 900,000 new cases of PCa are diagnosed worldwide, constituting approximately 14% of all cancer cases.^[4,5]^ Consequently, the diagnosis and treatment of PCa pose substantial challenges.

Given these risk factors, the association between prostate cancer and DNA damage repair (DDR) pathways, crucial for genomic stability, has become a focal point in recent research.

**Figure 1.**
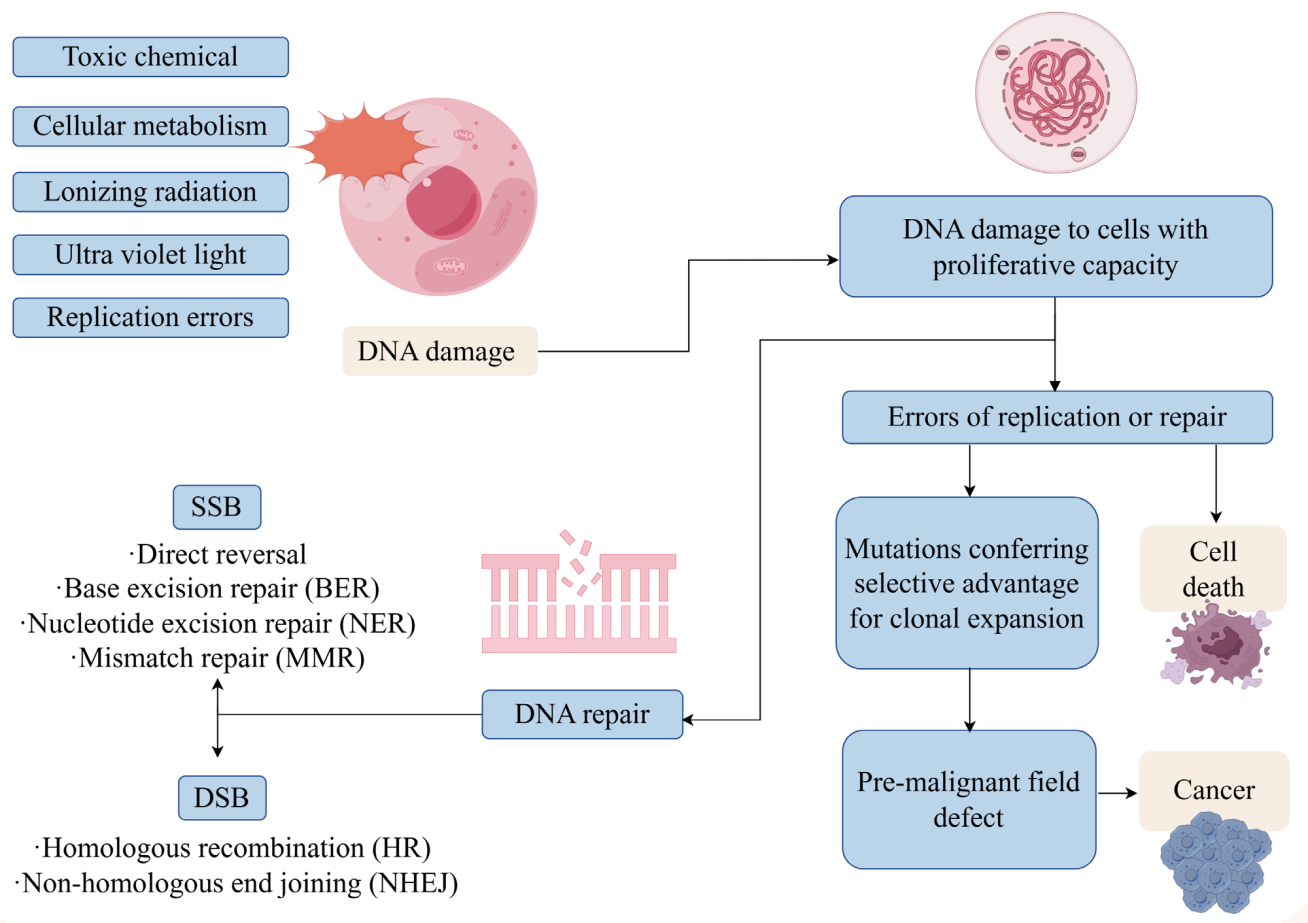
DNA damage repair and DNA damage repair pathways. When DNA is damaged, cells initiate a series of DNA damage repair processes, sensing and prompting DNA damage, signaling, and promoting subsequent repair (By Figdraw).

Cellular DNA damage is an inevitable occurrence, resulting in either single-strand breaks (SSBs) or double-strand breaks (DSBs), both of which necessitate accurate and timely repair to maintain genomic stability.^[6–8]^ SSBs are typically managed by base excision repair (BER), nucleotide excision repair (NER), and mismatch repair (MMR) pathways, while DSBs are addressed through homologous recombination (HR) or nonhomologous end joining (NHEJ)^[9–13]^ (Fig. 1). The genetic susceptibility to PCa has been linked to rare germline mutations in DNA damage repair (DDR) genes, with the prognosis and aggressiveness of the disease often associated with aberrant DDR activation or inactivation.^[14]^ DDR is essential in preserving genomic stability, and its dysfunction can lead to genomic instability and promote tumorigenesis in various cancers. Elevated mutation rates in DDR genes have been observed in PCa patients,^[14,15]^ along with numerous genomic alterations related to DNA repair in cases of metastatic castration-resistant prostate cancer (mCRPC), highlighting potential avenues for targeted molecular therapies.^[16]^ While the role of DDR in PCa pathogenesis is evident, the mechanisms remain incompletely understood, warranting further investigation.

The Mendelian Randomization (MR) method, which uses genetic variation to assess causal relationships, effectively avoids the interference of unknown confounding factors and reverse causality.^[17]^ This method offers a robust tool to investigate the association between the DDR-related genes and PCa, uncovering intricate genetic interactions and disease mechanisms, and identifying novel targets for early diagnosis, risk evaluation, and treatment of PCa.

## 2. Methods

**Figure 2.**
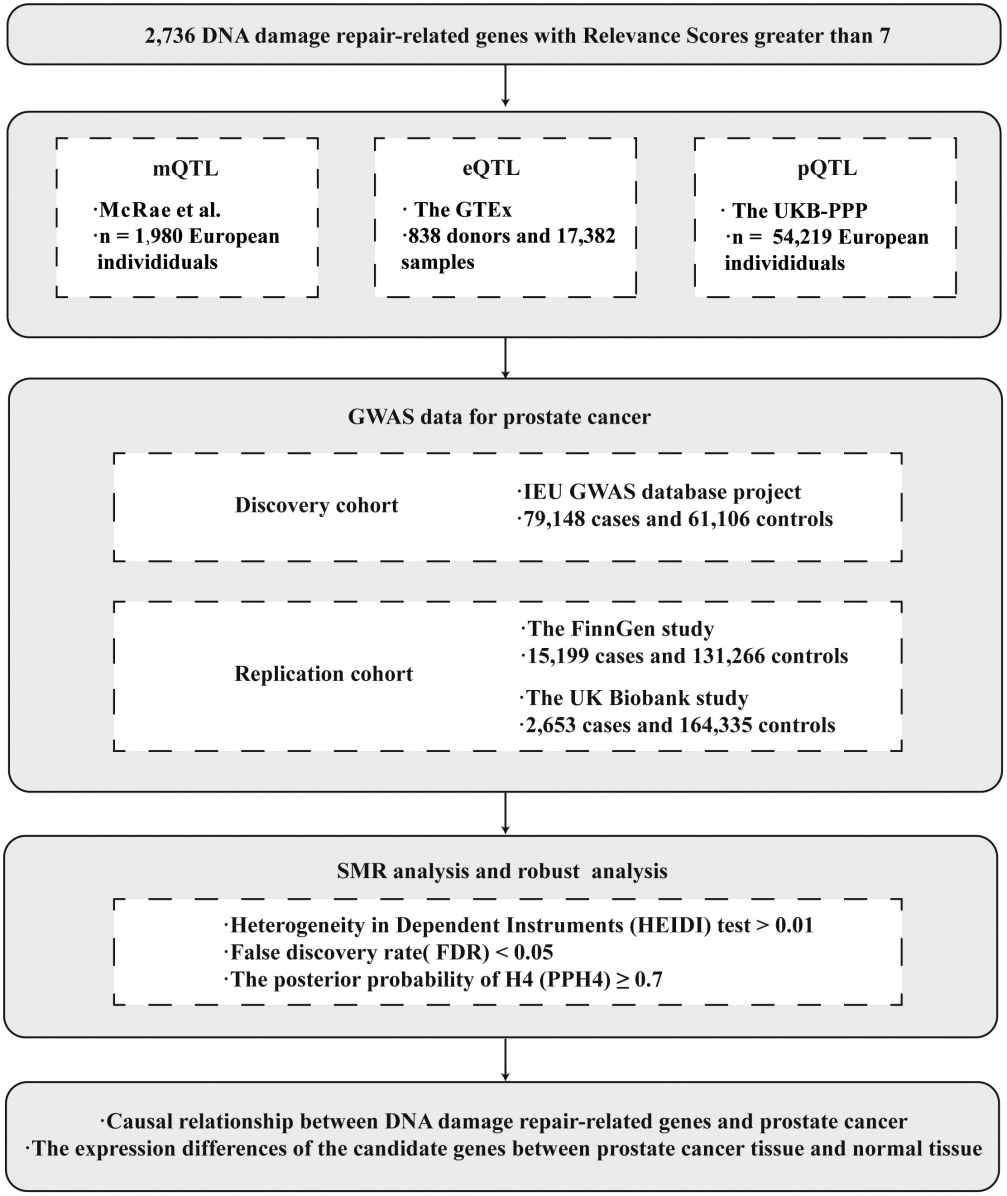
The workflow of this study. QTL = quantitative trait loci, GTE = genotype tissue expression, UKB-PPP = UK Biobank pharma proteomics project; GWAS = Genome-wide association study, SMR = summary-based Mendelian randomization, HEIDI = heterogeneity in dependent instruments, FDR = false discovery rate.

This study workflow is shown in Figure 2. Several quantitative trait loci (QTLs) were analyzed, including gene methylation (mQTLs), gene expression (eQTLs), and protein abundance (pQTLs). PCa summary-data were obtained from Genome-Wide Association Studies (GWAS). We conducted our analysis using the Summary-data-based Mendelian Randomization (SMR) method. We further investigated the expression of candidate genes in PCa tissue using the Human Protein Atlas (HPA) database and the Tumor Immune Single-cell Hub 2 (TISCH2) database.

### 2.1. Data sources

We searched the GeneCards database for DDR-related genes using the keyword “DNA damage repair” and included genes with a relevance score of 7 or higher in the analysis,^[18,19]^ resulting in a total of 2736 DDR-related genes (Table S1). We obtained mQTL summary-data from a meta-analysis by McRae et al, which incorporated 2 European cohorts: the Brisbane Systems Genetics Study (n = 614) and the Lothian Birth Cohorts (n = 1366). Whole-blood methylation data were measured using the Illumina HumanMethylation450 array.^[20]^ We obtained eQTL data from the Genotype-Tissue Expression (GTEx) portal (https://gtexportal.org/). The GTEx V8 dataset includes 838 donors and 17,382 samples across 52 tissues and 2 cell lines.^[21]^ Our study utilized eQTL data from whole blood and validated it with data from prostate-specific tissues. The pQTL data were derived from the UK Biobank Pharmacogenomics Project (UKB-PPP), which analyzed 2941 plasma analytes from 54,219 UKB participants.^[22]^ The GWAS data for PCa were based on publicly available datasets, with the discovery cohort originating from the International Epidemiology United Kingdom (IEU) GWAS database project.^[23]^ The replication cohorts were from the UK Biobank^[24]^ and the FinnGen study.^[25]^ The discovery cohort comprised 79,148 cases and 61,106 controls. Replication cohorts from the UK Biobank included 2653 cases and 164,335 controls, while the FinnGen study R10 data included 15,199 cases and 131,266 controls. All data were derived from European populations, with no sample overlap between the datasets. We analyzed candidate gene expression levels using the Human Protein Atlas (HPA) database (https://www.proteinatlas.org/). For single-cell expression analysis in PCa tissue, we utilized the GSE137829 PCa dataset from the TISCH2 database (https://tisch.compbio.cn/home/) (Table S2). This study involved a secondary analysis of publicly available, de-identified data and therefore did not require additional ethical review or informed consent. The original studies from which the data were derived had obtained approval from the appropriate institutional ethics committees, and the relevant ethical information can be found in their published reports.

### 2.2. Data analysis

The study primary analytical process comprised 2 stages: SMR analysis and robust analysis. During the SMR analysis phase, our approach involved analyzing multiple single nucleotide polymorphisms (SNPs). SMR can achieve greater statistical power than traditional MR analysis when the exposure and outcome are derived from 2 independent samples with large sample sizes.^[26]^ We selected the optimal cis-QTL within a window of ± 1000 kb centered on the corresponding gene and used a *P*-value threshold of 5.0 × 10^-8^. We filtered out SNPs with allele frequency differences exceeding a threshold of 0.2 across different datasets. To distinguish between pleiotropy and linkage, we utilized the Heterogeneity in the dependent instrument (HEIDI) test, excluding those with a *P*-HEIDI value <.01, indicative of pleiotropy. We applied the Benjamini-Hochberg method to adjust *P*-values, ensuring a false discovery rate (FDR) of <.05. The robust analysis employed a collocation analysis approach to detect shared causal variants. The collocation analysis assigned 5 different posterior probabilities to corresponding hypotheses: No trait has a causal variant (H0); Only gene expression has a causal variant (H1); Only disease risk has a causal variant (H2); The 2 traits have different causal variants (H3); The 2 traits share the same causal variant (H4). Colocalization analysis was conducted between QTL and GWAS data using collocation region windows set to ± 1000 kb, ±1000 kb, and ± 500 kb, respectively. A posterior probability of H4 (PPH4) ≥ 0.7 indicates evidence of collocation between GWAS and QTL.^[27]^ The SMR and HEIDI tests were conducted using the SMR software tool (SMR v1.3.1), while the collocation analysis utilized the coloc R package (R v4.3.2).

### 2.3. Data integration

To deepen our comprehension of the possible causal association between DDR- related genes and PCa, we synthesized findings across 3 distinct levels of gene regulation. Candidate genes were identified based on significant associations with PCa across at least 2 of the following: gene methylation, gene expression, and protein abundance levels (adjusted *P* values for FDR < .05 and PPH4 ≥ 0.7 in the collocation analysis). To further explore the potential regulatory relationship between candidate genes and PCa, we validated these genes using eQTL data from prostate-specific tissues. Finally, we analyzed the expression differences of the candidate genes between prostate cancer tissue and normal tissue.

## 3. Results

### 3.1. mQTL and GWAS data

The impact of DDR-related gene methylation on PCa is detailed in Table S3. Following FDR correction and exclusion of data with *P*-HEIDI < .01, robustness analysis with collocation (PPH4 ≥ 0.7) identified a total of 136 CpG sites across 44 distinct genes. Among these, 54 CpG sites from 21 unique genes were validated in the FinnGen study, and 60 CpG sites from 18 unique genes were validated in the UK Biobank study (Table S4).

### 3.2. eQTL and GWAS data

The findings for the causal effects of DDR-related gene expression on PCa are detailed in Table S5. Following correction for multiple testing and collocation analysis, 11 genes exhibited significant associations with PCa (adjusted *P* < .05). Higher genetically predicted expression levels of CASP8 (OR 1.163, 95%CI 1.096–1.235; PPH4 = 0.963), LMNA (OR 1.178, 95%CI 1.065–1.303; PPH4 = 0.715), NUCKS1 (OR 1.563, 95% CI 1.269–1.925; PPH4 = 0.954), RTEL1 (OR 1.557, 95%CI 1.257–1.927; PPH4 = 0.985), and ZBTB38 (OR 1.310, 95%CI 1.166–1.471; PPH4 = 0.958) showed positive correlations with PCa risk. Conversely, higher genetically predicted expression levels of COL11A2 (OR 0.898, 95%CI 0.845–0.955; PPH4 = 0.962), KMT2B (OR 0.723, 95%CI 0.603–0.867; PPH4 = 0.894), POLI (OR 0.827, 95% CI 0.764–0.895; PPH4 = 0.941), RPA2 (OR 0.945, 95%CI 0.916–0.976; PPH4 = 0.832), SREBF1 (OR 0.910, 95%CI 0.873–0.949; PPH4 = 0.946), and TRIM26 (OR 0.695, 95%CI 0.597–0.809; PPH4 = 0.849) showed negative correlations with PCa risk. The associations of LMNA, POLI, and RPA2 were replicated in the FinnGen study. Additionally, the associations of NUCKS1, ZBTB38, and SREBF1 were replicated in the UK Biobank study (Table S6).

### 3.3. pQTL and GWAS data

The findings concerning the causal impacts of DDR-related protein abundance on PCa are detailed in Table S7. Following correction for multiple testing and collocation analysis, higher genetically predicted expression levels of RPA2 (OR 0.714, 95%CI 0.589–0.866; PPH4 = 0.749) were inversely associated with PCa risk. This association of RPA2 was also validated in the FinnGen study (refer to Table S8).

### 3.4. Multi-omics data integration

**Figure 3.**
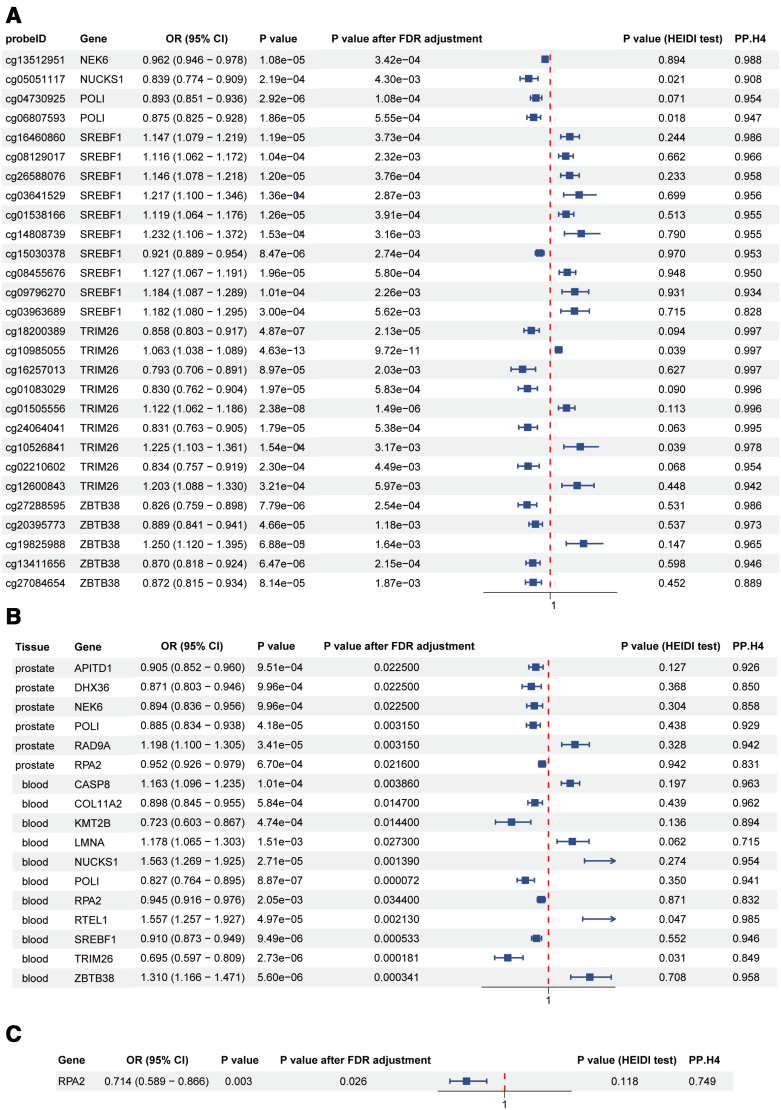
Associations of genetically predicted gene methylation (A), gene expression (B), and protein abundance (C) with prostate cancer in Mendelian randomization analysis. FDR = false discovery rate, HEIDI = heterogeneity in dependent instruments, SNP = single nucleotide polymorphism, OR = odds ratio; CI = confidence interval.

**Figure 4.**
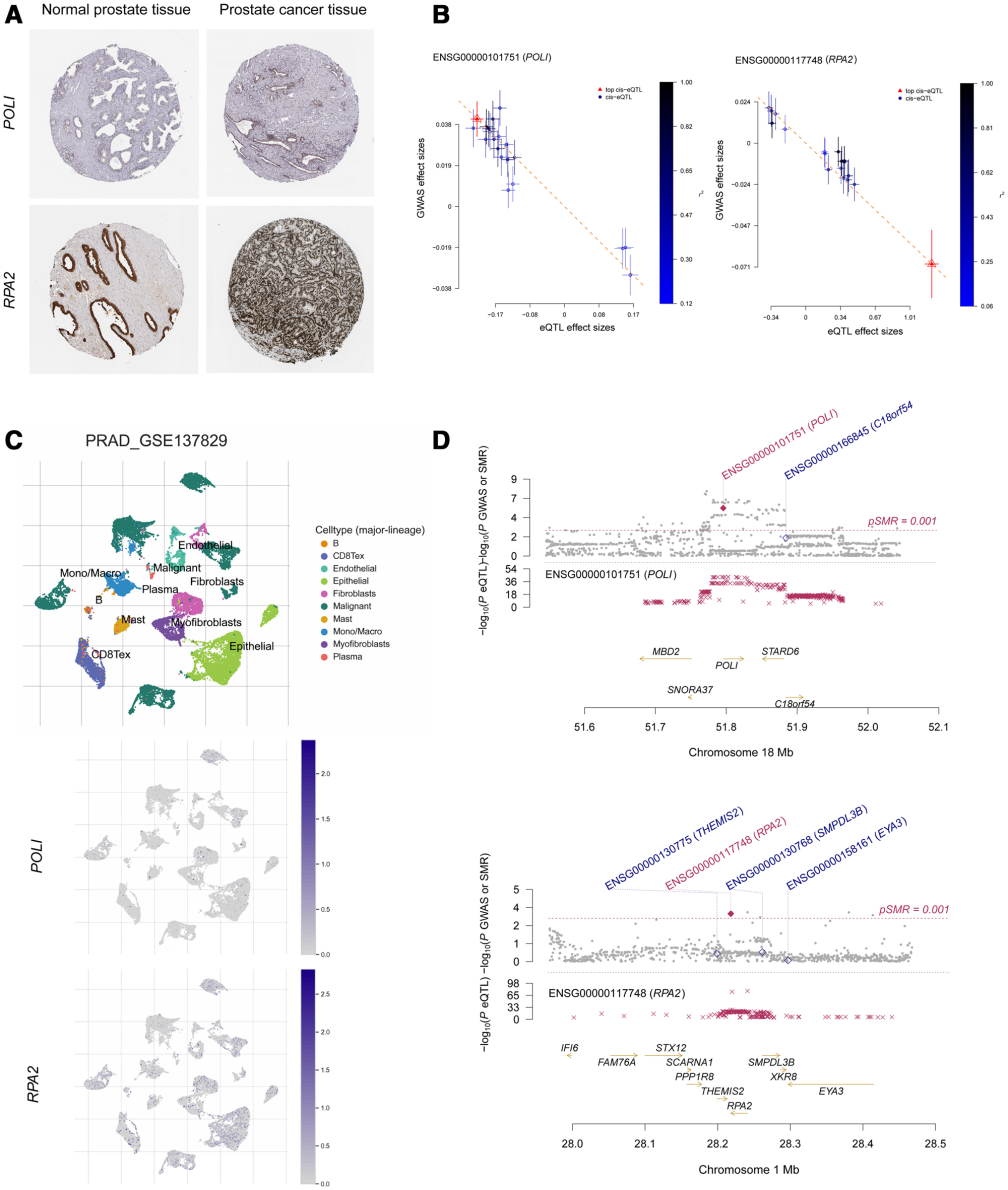
(A) Protein abundance profiling of POLI and PRA2 in prostate cancer and adjacent normal prostate tissue. (B) Scatter plot of SMR analysis. Linear regression analyses of the effect values of the included SNPs in the eQTL and GWAS data were performed to demonstrate the associations between the expression levels of the genes represented by the eQTLs and the traits studied in the GWAS. (C) UMAP plots displaying the clustering of different cell types, Single-cell expression levels of POLI and RPA2 in prostate cancer tissue. (D) Regional plot of SMR analysis. Layer 1 shows the gene expression probes that passed the SMR analysis, using red to indicate that the probe passed the SMR threshold, and solid to indicate the genes that passed the HEIDI test. Layer 2 shows the chromosomal base position and *P*-value of the SNP corresponding to the gene. Layer 3 shows the corresponding gene information within the chromosomal interval. SMR = Summary-based Mendelian Randomization; SNP = Single Nucleotide Polymorphism; eQTL = Expression Quantitative Trait Loci; GWAS = Genome-Wide Association Study; HEIDI = Heterogeneity In Dependent Instruments; UMAP = Uniform Manifold Approximation and Projection.

By integrating results from the 3 different regulatory levels, a total of 7 genes met our selection criteria: NUCKS1, POLI, RPA2, RTEL1, SREBF1, TRIM26, ZBTB38, and NEK6 (Fig. 3). Among these, POLI (OR 0.885, 95%CI 0.834–0.938; PPH4 = 0.929) and RPA2 (OR 0.952, 95%CI 0.926–0.979; PPH4 = 0.831) were validated in prostate tissue-specific eQTL data. The HPA database confirmed the expression of RPA2 and POLI proteins in prostate cancer tissue. Additionally, single-cell uniform manifold approximation and projection (UMAP) analysis from the TISCH2 database revealed the expression of POLI and RPA2 in various cell types including B cells, CD8 + T cells, endothelial cells, epithelial cells, fibroblasts, malignant cells, mast cells, monocytes/macrophages, myofibroblasts, and plasma cells within prostate cancer tissue (Fig. 4).

## 4. Discussion

In this study, we performed SMR analyses at the methylation, gene expression, and protein abundance levels to investigate the association between genetically predicted DDR-related genes and PCa. Our multi-omics evidence suggests that POLI and RPA2 may be associated with the risk of PCa. Analysis of immunohistochemical results confirmed the expression of POLI and RPA2 in prostate cancer tissue. Interestingly, we found that single-cell sequencing data from tumor tissue showed higher expression of RPA2 compared to POLI, with both expressed in malignant cell subpopulations, further indicating a close relationship between POLI and RPA2 and PCa.

Replication Protein A (RPA) plays a central role in essential DNA metabolic processes that are critical for maintaining genomic stability.^[28]^ Earlier research has indicated that RPA2 participates in DNA replication and repair processes by interacting with multiple proteins and demonstrating a high affinity for both single-stranded DNA (ssDNA) and double-stranded DNA (dsDNA). Its functions encompass DNA replication, repair, recombination, and telomere maintenance.^[29]^ During DNA replication, RPA2 may participate in the remodeling of the replication fork, particularly when encountering ssDNA regions, by coordinating the activity of uracil-N-glycosylase (UNG) and other proteins through its winged-helix (WH) domain to maintain the integrity of DNA replication and prevent mutations.^[30]^ In DNA damage, hyperphosphorylation of RPA2 can prevent the normal recruitment of DNA repair proteins such as RAD51 and the PP4 protein phosphatase complex to DNA, affecting the repair of DNA damage and the maintenance of the replication fork, thereby regulating its role in various genotoxic stress responses.^[31,32]^ Previous studies have indicated that BRCA1 and BRCA2 are closely associated with the development and progression of prostate cancer. The BRCA proteins play a pivotal role in DNA replication and repair, and in cells with BRCA deficiency, more ssDNA gaps are produced during DNA replication. RPA2 marks these ssDNA gaps in BRCA-deficient cells, recruits SLFN11 to the gaps, and under the influence of poly ADP-ribose polymerase (PARP) inhibitors, can lead to DNA damage that may ultimately result in cell death.^[33]^ BRCA1 and RPA2 have a close functional connection in the repair process during DNA replication fork stalling and collapse. BRCA1 may regulate the HR repair mechanism by affecting the phosphorylation of RPA2 and the formation of ssDNA.^[34]^ The critical interaction between RPA2 and RAD52 in BRCA2-deficient cells for HR suggests that the dysfunction of RPA2 may further exacerbate BRCA-related defects and affect DNA repair.^[35]^ Further research has found that CBX3 may be a key regulator of RPA2 function and DNA replication. An increase in CBX3 causes RPA2 to be retained at the replication fork, which increases replication stress and DNA damage, thereby enhancing the response of cancer cells to PARP inhibitors and affecting the progression of prostate cancer and their response to treatment^[36]^ As a result, genetic mutations or downregulation of expression could lead to deficiencies in the repair of DSBs in DNA, which in turn increases genomic instability and is associated with the development of prostate cancer.

POLI is a Y-family DNA polymerase in humans that plays an essential role in the DNA replication process, especially in translesion synthesis (TLS) across damaged sites when DNA is damaged, helping to maintain the continuity of DNA replication.^[37]^ Studies have found that POLI has a unique role in regulating the DNA damage tolerance pathway, and when POLI function is impaired, it leads to PrimPol-dependent resumption, which accelerates DNA replication but simultaneously reduces replication stress signals and checkpoint activation during the S phase, leading to chromosomal instability during the M phase.^[38]^ Research has found that p53 can form a complex with POLI, triggering DNA damage tolerance, controlling the rate of DNA replication, and helping to process damage more accurately during replication.^[39]^ This complex may also temporarily stabilize the replication fork at replication obstacles through an “idling” mechanism, promoting the polyubiquitination of proliferating cell nuclear antigen, and facilitating damage bypass mechanisms and replication fork restart through HLTF and ZRANB3.^[40]^ The fusion of TMPRSS2 with ETS oncogenes frequently occurs in PCa. Notably, previous research has demonstrated a significant association between the F532S variant in the POLI gene and PCa positivity for the TMPRSS2-ERG fusion.^[41]^ This finding is congruent with our research results. Given that POLI is involved in the repair of DNA damage, especially when the replication fork encounters DNA damage, its loss of function may fail to effectively resolve stalled replication forks, leading to an increase in double-strand breaks in DNA. This could potentially facilitate chromosomal translocation events, such as the TMPRSS2-ERG fusion, which are crucial in the development of PCa. Understanding these mechanisms may contribute to a more comprehensive elucidation of the molecular pathogenesis of prostate cancer and offer potential targets for future therapeutic strategies.

The DDR mechanism plays a crucial role in maintaining genomic stability and preventing the occurrence of tumors, as it can release signals to activate an immune response, facilitating the recognition and attack of cancer cells by the immune system.^[42]^ Due to defects in DNA damage and repair, tumor cells may express new or altered antigens and Danger-Associated Molecular Patterns, which can activate dendritic cells and subsequently promote the activation and proliferation of T cells. This process involves the exposure of the cGAS-STING pathway.^[43,44]^ The activation of the cGAS-STING pathway can lead to immunogenic cell death, releasing tumor antigens, enhancing dendritic cell activation, and initiating T cell responses, thereby strengthening the immune response against the tumor. Deficiencies in the DNA mismatch repair mechanism can produce many mutations, which the immune system can recognize as nonself components, potentially generating new tumor-specific antigens that can stimulate an immune response and thus exert antitumor effects. However, the mechanisms of RPA2 and POLI in prostate cancer remain unreported, representing a key focus for future research.

In summary, our study found that higher expression of RPA2 and POLI is linked to a reduced risk of PCa. By integrating multi-omics with GWAS data, we systematically evaluated the causal relationships between 2736 DDR-related genes and PCa, with strict quality control ensuring robust and reliable findings.

The limitations of this study are as follows: First, the sample is restricted to individuals of European descent, which may limit the generalizability of our findings. Future research should include more diverse ethnic groups to enhance the applicability of the results. Second, the SMR method typically relies on single or limited genetic variations, and our analysis is confined to the cis-QTL data of DDR-related genes. This may not fully capture the complex biological mechanisms involved, necessitating functional experiments to validate our findings and explore deeper mechanisms. Lastly, we have only preliminarily investigated the interaction between the DDR mechanism and the immune system. Further research is required to elucidate the mechanisms by which related genes contribute to immune responses and to develop novel therapeutic strategies aimed at improving the quality of life and survival rates of prostate cancer patients.

## 5. Conclusion

This MR study investigated the potential causal roles of DNA Damage Repair (DDR)-related genes in the onset of prostate cancer (PCa) by examining methylation, gene expression, and protein abundance. The results underscore the significance of DDR-related genes RPA2 and POLI in PCa onset, providing new insights into the molecular mechanisms driving PCa and highlighting potential targets for pharmacological intervention.

## Acknowledgments

We extend our gratitude to the participants and researchers of the McRae et al genome-wide association study, the GTEx project, the UKB-PPP project, the IEU GWAS database project, the FinnGen study, the Human Protein Atlas, the Tumor Immune Single-cell Hub 2 and the UK Biobank study for providing data.

## Author contributions

**Conceptualization:** Huadong Xie, Chengjie Ban, Xianlin Yi.

**Methodology:** Huadong Xie, Chengjie Ban, Zhengwei Su, Qingming Huang, Han Tang, Zhi Cheng, Tianling Liao, Kangji Liao.

**Software:** Huadong Xie, Chengjie Ban.

**Validation:** Huadong Xie, Chengjie Ban.

**Visualization:** Huadong Xie.

**Writing – original draft:** Huadong Xie, Chengjie Ban.

**Writing – review & editing:** Huadong Xie, Chengjie Ban, Xianlin Yi.

**Data curation:** Zhengwei Su, Qingming Huang, Han Tang, Zhi Cheng, Tianling Liao, Kangji Liao.

**Investigation:** Zhengwei Su, Qingming Huang, Han Tang, Zhi Cheng, Tianling Liao, Kangji Liao.

**Funding acquisition:** Xianlin Yi.

**Supervision:** Xianlin Yi.
















